# Digital cognitive behaviour therapy for insomnia (dCBT‐I): Chronotype moderation on intervention outcomes

**DOI:** 10.1111/jsr.13572

**Published:** 2022-02-27

**Authors:** Patrick Faaland, Øystein Vedaa, Knut Langsrud, Børge Sivertsen, Stian Lydersen, Cecilie L. Vestergaard, Kaia Kjørstad, Daniel Vethe, Lee M. Ritterband, Allison G. Harvey, Tore C. Stiles, Jan Scott, Håvard Kallestad

**Affiliations:** ^1^ Department of Mental Health Norwegian University of Science and Technology Trondheim Norway; ^2^ St Olavs University Hospital, Østmarka Trondheim Norway; ^3^ Department of Health Promotion Norwegian Institute of Public Health Bergen Norway; ^4^ Voss District Psychiatric Hospital NKS Bjørkeli Voss Norway; ^5^ Department of Research and Development St Olavs University Hospital Trondheim Norway; ^6^ Department of Research and Innovation Fonna Health Trust Haugesund Norway; ^7^ Department of Mental Health Regional Centre for Child and Youth Mental Health and Child Welfare Norwegian University of Science and Technology Trondheim Norway; ^8^ Center for Behavioral Health and Technology Department of Psychiatry and Neurobehavioral Sciences University of Virginia Charlottesville Virginia USA; ^9^ Department of Psychology University of California Berkeley California USA; ^10^ Department of Psychology Norwegian University of Science and Technology Norway; ^11^ University of Newcastle Newcastle UK

**Keywords:** chronotype, circadian preferences, digital cognitive therapy for insomnia, insomnia, moderator, randomized‐controlled trial

## Abstract

Using data from 1721 participants in a community‐based randomized control trial of digital cognitive behavioural therapy for insomnia compared with patient education, we employed linear mixed modelling analyses to examine whether chronotype moderated the benefits of digital cognitive behavioural therapy for insomnia on self‐reported levels of insomnia severity, fatigue and psychological distress. Baseline self‐ratings on the reduced version of the Horne–Östberg Morningness–Eveningness Questionnaire were used to categorize the sample into three chronotypes: morning type (*n* = 345; 20%); intermediate type (*n* = 843; 49%); and evening type (*n* = 524; 30%). Insomnia Severity Index, Chalder Fatigue Questionnaire, and Hospital Anxiety and Depression Scale were assessed pre‐ and post‐intervention (9 weeks). For individuals with self‐reported morning or intermediate chronotypes, digital cognitive behavioural therapy for insomnia was superior to patient education on all ratings (Insomnia Severity Index, Chalder Fatigue Questionnaire, and Hospital Anxiety and Depression Scale) at follow‐up (*p*‐values ≤ 0.05). For individuals with self‐reported evening chronotype, digital cognitive behavioural therapy for insomnia was superior to patient education for Insomnia Severity Index and Chalder Fatigue Questionnaire, but not on the Hospital Anxiety and Depression Scale (*p* = 0.139). There were significant differences in the treatment effects between the three chronotypes on the Insomnia Severity Index (*p* = 0.023) estimated difference between evening and morning type of −1.70, 95% confidence interval: −2.96 to −0.45, *p* = 0.008, and estimated difference between evening and intermediate type −1.53, 95% confidence interval: −3.04 to −0.03, *p* = 0.046. There were no significant differences in the treatment effects between the three chronotypes on the Chalder Fatigue Questionnaire (*p* = 0.488) or the Hospital Anxiety and Depression Scale (*p* = 0.536). We conclude that self‐reported chronotype moderates the effects of digital cognitive behavioural therapy for insomnia on insomnia severity, but not on psychological distress or fatigue.

## 1 INTRODUCTION

Cognitive behavioural therapy for insomnia (CBT‐I) is the recommended first‐line treatment for chronic insomnia (Riemann et al., 2017). Increasing evidence indicates that, like face to face CBT‐I, digital CBT‐I (dCBT‐I) is associated with large effects for improvement across a range of sleep, psychological and functional outcomes for individuals with insomnia (Zachariae, Lyby, Ritterband, & O'Toole, 2016). However, 30%–60% of individuals offered dCBT‐I do not demonstrate clinically significant benefits over and above those attained with a comparator intervention, such as patient education about sleep (PE; Blom et al., 2015; Ritterband et al., 2009; Vedaa et al., 2020). Accordingly, identifying factors that moderate outcomes for dCBT‐I may help determine who might most benefit from this intervention, and how it might be targeted at populations most likely to attain positive outcomes (Luik, van der Zweerde, van Straten, & Lancee, 2019). This is particularly important if individuals are given direct access to dCBT‐I, and situations where pre‐intervention screening is undertaken using self‐report rather than clinical assessment. One major strength with moderator analysis within a randomized controlled trial (RCT) is to identify subgroups of individuals within the population who might respond differently to an intervention (e.g. according to factors such as sex, age, socioeconomic status, ethnicity). However, large sample sizes are needed to show reliable interactions between the moderator and the interventions that are being compared (Kraemer, Frank, & Kupfer, 2006).

To date, evidence indicates that CBT‐I can be efficacious for individuals with insomnia and a range of comorbid conditions (Zachariae et al., 2016). There is no consistent evidence that socio‐demographic variables moderate outcomes of dCBT‐I (Cheng et al., 2019). A plausible moderator for CBT‐I might be chronotype, due to differences in: clinical presentation (i.e. fatigue, psychological distress and depressive symptoms; Bei et al., 2015; Lien et al., 2019); individuals with different chronotypes also show differences in sleep–wake measures (i.e. sleep–wake measures, sleep–wake profiles, self‐reported sleep quality, daytime sleepiness, sleep‐onset latency (SOL), sleep variability and daytime activity; Barclay, Eley, Buysse, Archer, & Gregory, 2010; Giannotti, Cortesi, Sebastiani, & Ottaviano, 2002; Natale & Cicogna, 2002; Taillard, Philip, & Bioulac, 1999; Thun et al., 2012; Yazdi, Sadeghniiat‐Haghighi, Javadi, & Rikhtegar, 2014); and measures of circadian phase (i.e. timing of core body temperature, melatonin excretion and cortisol profiles; Baehr, Revelle, & Eastman, 2000; Bailey & Heitkemper, 2001; Gibertini, Graham, & Cook, 1999). Individual differences in both clinical, sleep‐related and biological measures might result in different insomnia symptoms and response to CBT‐I.

To our knowledge, few studies have explored the potential impact of circadian preference (i.e. chronotype) on outcomes of dCBT‐I (Asarnow et al., 2019; Bei, Ong, Rajaratnam, & Manber, 2015; Lien et al., 2019), despite differences in the clinical presentation (Bei et al., 2015; Lien et al., 2019). Chronotype differences indicate a somewhat different response on depressive symptoms after CBT‐I (Asarnow et al., 2019; Bei et al., 2015). Previous studies have all divided subgroups differently, and within different populations of participants. Use of standardized instruments with predefined ranges to create subgroups may provide a better basis for comparing findings across studies. Taken together, there are indications that individuals with insomnia may differ on key clinical and sleep variables depending on their chronotype. However, findings are inconsistent regarding the association between chronotype and clinical outcome with CBT‐I.

### 1.1 Aims

This study represents a planned analysis of data from a recently published RCT on the effects of dCBT‐I compared with PE in a large‐scale, community‐based sample of 1721 Norwegian adults with self‐reported insomnia (Vedaa et al., 2020). The primary aim is to test if self‐reported chronotype moderates between‐group differences in levels of insomnia severity at 9‐week follow‐up. The secondary aims are to test if chronotype moderates between‐group differences in levels of fatigue and psychological distress at 9‐week follow‐up. Additionally, we examined whether any differences between baseline ratings of demographic, sleep–wake patterns, and levels of insomnia severity, fatigue and psychological distress are associated with different chronotypes.

## 2 METHODS

### 2.1 Design

Details of the study protocol, procedures and key outcomes are published elsewhere (Kallestad et al., 2018; Vedaa et al., 2020). The trial is registered on clinicaltrials.gov (registration number: NCT02558647), and was approved by the Regional Committee for Medical and Health Research in South‐East Norway (2015/134) and followed the CONSORT guidelines (Moher et al., 2010).

### 2.2 Participants

Individuals with self‐identified sleep problems were recruited from February 2016 to July 2018 through postings in general practitioners' offices, advertisements by Norwegian University of Science and Technology, the Norwegian Institute of Public Health, and via various media. Potential participants were directed to a website to complete an online informed consent and screening measure. Inclusion criteria were: age ≥ 18 years and score ≥ 12 on the Insomnia Severity Index (ISI; Filosa et al., 2020). Exclusion criteria were: scored ≥ 11 on the Epworth Sleepiness Scale, and/or reported excessive snoring, breathing stops or having difficulties staying awake during the day; other self‐reported medical conditions that may be a contra‐indication for CBT‐I (e.g. recent myocardial infarction); and/or being engaged in shift or night work. Below, key methodological details for the RCT are summarized (further details are provided elsewhere; Kallestad et al., 2018).

### 2.3 Procedure

After screening, eligible participants completed a range of baseline assessments, including sleep diary self‐ratings (at least 10 days of ratings over two consecutive weeks). Participants were subsequently randomized to either dCBT‐I or PE. All participants completed the same self‐reported assessments at 9‐week follow‐up (i.e. 9 weeks after randomization).

#### 2.3.1 dCBT‐I

Sleep Healthy Using the Internet (SHUTi) is a fully automated and interactive web‐based program that incorporates primary strategies and techniques from CBT‐I (Ritterband et al., 2009). This includes sleep restriction, stimulus control, cognitive restructuring, sleep hygiene and relapse prevention. The program is individually adapted, sets learning objectives and performance requirements, and provides feedback on intervention achievements and targets. The program consists of six cores that become available 7 days post‐completion of the previous one. Each core consists of objectives, activity review, feedback, new content and homework. Users also track their sleep by entering their sleep diary data into the system to enable a more tailored intervention program.

#### 2.3.2 Patient education

The PE group received access to a website containing patient information related to sleep, including basic CBT‐I principles. The PE website consisted of static text all made available immediately and could be accessed throughout the study period. The participants in the PE condition were encouraged to visit the website at the start of the study, without further notifications. While both the dCBT‐I and PE condition provided sleep diaries, the PE site did not offer online tools for self‐monitoring or feedback.

### 2.4 Assessments utilized in the moderator analyses

For the purposes of this study, we extracted data from baseline and 9‐week (post‐intervention) follow‐up assessments for the following measures.

#### 2.4.1 The Horne–Östberg Morningness–Eveningness Questionnaire, reduced version (rMEQ)

The rMEQ is an abbreviated version of the Horne–Östberg Morningness–Eveningness Questionnaire containing five items. The composite scores range from 4 to 25, with lower scores indicating greater preference for eveningness (Adan & Almirall, 1991). Based on self‐ratings, individuals were classified into three categories: evening type were those who scored between 4 and 11; intermediate types scored between 12 and 17; and morning types scored between 18 and 25. Additionally, individuals were divided into five subgroups as rMEQ is clinically scored, and to better differentiate between the extremes in circadian preference subtypes. The five groups were divided into: definitely evening type, scores between 4 and 7; moderately evening type, scores between 8 and 11; neither type, scores between 12 and 17; moderately morning type, scores between 18 and 21; and definitely morning type, scores between 22 and 25 (Table S2).

#### 2.4.2 Insomnia Severity Index

The ISI consists of seven items that specify the participants' overall insomnia severity (Bastien, Vallieres, & Morin, 2001). The items are rated on a 5‐point Likert scale from 0 to 4, with total scores ranging from 0 to 28. Higher scores indicate greater insomnia severity. The ISI was assessed at baseline and 9‐week follow‐up. The ISI has good psychometric properties (Bastien et al., 2001).

#### 2.4.3 Chalder Fatigue Questionnaire (CFQ)

The CFQ consists of 13 items addressing physical and psychological fatigue, including items addressing duration and intensity of fatigue complaints. Each item is scored on a 4‐point scale ranging from asymptomatic to maximum symptomatology. A composite score is calculated by combining the 13 items, with a total fatigue scale ranging from 0 to 39 points, higher scores indicate more fatigue symptoms (Chalder et al., 1993). The CFQ was assessed at baseline and 9‐week follow‐up.

#### 2.4.4 Hospital Anxiety and Depression Scale (HADS)

The HADS consists of 14 items that assess symptoms of anxiety and depression. The items are rated on a 4‐point Likert scale from 0 to 3, with a range from 0 to 42 (Zigmond & Snaith, 1983). The total score was used as a measure of psychological distress, with higher scores indicating more psychological distress. The HADS was administered at baseline and 9‐week follow‐up.

#### 2.4.5 Consensus sleep diary

The consensus diary includes 11 questions concerning bedtime, sleep onset, number and length of awakenings during the night, time of final awaking, rise time, number and length of daytime naps, use of medication and alcohol consumption (Carney et al., 2012). The sleep parameters derived from the diary were: wake after sleep onset (WASO); SOL; sleep efficiency (SE); early morning awakening (EMA); time in bed (TIB); and total sleep time (TST). Participants completed 10 diaries within a 14‐day period at baseline and 9‐week follow‐up.

### 2.5 Statistical analysis

Descriptive statistics at baseline are reported as means and standard deviations. Chi‐squared test and one‐way between group analysis of variance (ANOVA) were used to investigate baseline differences on demographic (i.e. age, sex, education level), sleep diary (i.e. SOL, WASO, EMA, SE, TST, TIB) and clinical measurements (i.e. ISI, CFQ, HADS) between the three chronotypes (i.e. rMEQ subgroups), and Tukey post hoc comparisons were conducted. Differences in missing data between chronotypes at follow‐up were tested using the chi squared test.

Linear mixed models were used with the ISI, CFQ and HADS, one at a time, as dependent variables. The individual was included as a random effect. Time, group (dCBT‐I versus PE), and chronotypes were included as covariates as follows: main effect of time and chronotypes, the two‐way interactions group × time and time × chronotypes, and the three‐way interaction group × time × chronotypes. In this way, by omitting a (systematic) main effect of group (at baseline) and the interaction group × chronotypes (at baseline), we adjust for baseline as recommended (Twisk et al., 2018). All analyses were adjusted for age and sex. The three‐way interaction terms were used to test if the estimated mean difference between dCBT‐I and PE is different between the different chronotypes groups. The effect of dCBT‐I versus PE at 9‐week follow‐up for the outcome variables (ISI, CFQ, HADS) within each of the three chronotypes groups is estimated as the difference in change from baseline for the two groups in terms of the coefficient of the corresponding interaction term group × time. In a linear mixed model, participants with missing data at follow‐up contribute in the estimation with data from baseline in such a way that the results are unbiased under the missing at random (MAR) assumption, while a complete case analysis would have been unbiased only under the more restrictive missing completely at random (MCAR) assumption. Standardized effect sizes (Cohen's *d*) were calculated for the between‐group assessments using (estimate mean difference⁄pooled baseline SD). The same linear mixed models were used to test moderation on five chronotype groups (Table S2).

Normality of residuals was checked by visual inspection of QQ plots. Two‐sided *p*‐values less than 0.05 were regarded as statistically significant. Statistical analyses were conducted using SPSS 25.

## 3 RESULTS

### 3.1 Baseline characteristics

As shown in Table 1, 345 (20%) individuals were categorized as having a morning chronotype, 843 (49%) as intermediate chronotype, and 524 (30%) as evening chronotype. Age differed significantly across the three categories, individuals with evening chronotype were younger than morning and intermediate chronotypes (*p* < 0.001), sex did not differ between the chronotypes (*p* = 0.065), with the proportion of females per chronotype ranging from 64% to 70%. Years of education did not differ between chronotypes (*p* = 0.763).

**TABLE 1:** Baseline characteristics for chronotype groups

	Morning chronotype^a^ *n* = 345		Intermediate chronotype^b^ *n* = 843		Evening chronotype^c^ *n* = 524	
	Mean	SD	Mean	SD	Mean	SD
rMEQ score	19.3	1.35	14.6	1.70	8.7	1.97
Age (years)	47.7^b,c^	12.21	46.6^a,c^	14.46	39.3^a,b^	13.21
Education level (years)	16.3	2.95	16.2	2.85	16.3	2.98
SOL (min)	40.3^b,c^	33.32	54.7^a,c^	43.38	66.4^a,b^	51.23
WASO (min)	52.2^c^	37.22	49.4^c^	41.16	33.3^a,b^	33.63
EMA (min)	43.5^c^	35.47	45.0^c^	34.15	37.6^a,b^	40.80
SE	71.7^b^	12.31	70.6^c^	13.66	73.6^b^	13.26
TST (hr)	5.7^c^	1.09	6.2^c^	1.24	6.2^a,b^	1.27
TIB (hr)	7.9^b,c^	0.87	8.3^a,c^	0.93	8.5^a,b^	1.13
ISI	19.6	3.90	19.5	3.84	19.1	4.04
CFQ	19.7^b,c^	6.03	20.8^a,c^	5.93	21.7^a,b^	5.89
HADS‐total	12.4^c^	6.73	13.0^c^	6.99	14.5^a,b^	7.18

Superscripts denote the results of between‐group post hoc tests that indicate differences from morning chronotype,^a^ intermediate chronotype^b^ and evening chronotype.^c^ Significant differences = *p* < 0.05.

CFQ, Chalder Fatigue Questionnaire; EMA, early morning awakening; HADS, Hospital Anxiety and Depression Scale; ISI, Insomnia Severity Index; rMEQ, Horne–Östberg Morningness–Eveningness Questionnaire, reduced version; SE, sleep efficiency; SOL, sleep‐onset latency; TIB, time in bed; TST, total sleep time; WASO, wake after sleep onset.

All sleep diary metrics were significantly different between the chronotype groups: SOL (*p* < 0.001), WASO (*p* < 0.001), EMA (*p* < 0.001), SE (*p* < 0.001), TST (*p* < 0.001) and TIB (*p* < 0.001).

Mean ISI scores at baseline were not significantly different across the chronotypes (*p* = 0.083), whereas mean scores on the CFQ (*p* < 0.001) and the HADS (*p* < 0.001) were both significantly different: individuals classified as evening chronotype reported higher scores on the CFQ and HADS than individuals with morning and intermediate chronotype (see Table 1 for findings of post hoc analyses).

### 3.2 Moderator analyses

Descriptive data for each chronotype group at baseline and follow‐up are shown in Table 2. The percentage of missing data for participants in dCBT‐I from baseline to follow‐up was significantly different between the chronotype groups on ISI (*p* < 0.001), CFQ (*p* < 0.001) and HADS (*p* = 0.003), with the lowest response rate observed in the evening chronotype. There were no significant differences between chronotype groups in PE on ISI (*p* = 0.659), CFQ (*p* = 0.677) or HADS (*p* = 0.646).

**TABLE 2:** Primary and secondary outcomes at 9‐week follow‐up assessment for participants with different chronotypes who were allocated to either dCBT‐I (*n* = 867) or PE (*n* = 853)

		dCBT‐I					PE				Adjusted mean difference			
		*n*		%	Mean	SD	*n*	%	Mean	SD	Estimate	95% CI	*p*‐value	Cohen's d
ISI														
Morning chronotype	Baseline	163			19.48	4.01	179		19.70	3.83				
	9‐week Follow‐up	124		76	9.95	6.45	117	65	15.19	4.91	−5.25	−6.37 to −4.12	<0.001	1.34
Intermediate chronotype	Baseline	410			19.25	3.75	402		19.88	3.97				
	9‐week Follow‐up	293		72	9.97	6.13	250	62	15.50	5.36	−5.42	−6.16 to −4.67	<0.001	1.38
Evening chronotype	Baseline	253			18.89	4.01	234		19.12	4.04				
	9‐week Follow‐up	153		61	11.11	6.23	143	61	14.68	5.37	−3.71	−4.72 to −2.70	<0.001	0.95
CFQ														
Morning chronotype	Baseline	163			19.62	6.30	179		19.83	5.86				
	9‐week Follow‐up	121	74		14.38	7.38	116	65	16.60	6.58	−2.67	−4.12 to −1.22	<0.001	0.45
Intermediate chronotype	Baseline	410			20.56	5.95	401		21.11	6.02				
	9‐week Follow‐up	290	71		14.86	7.02	246	61	17.43	5.97	−2.59	−3.56 to −1.63	<0.001	0.43
Evening chronotype	Baseline		253		21.93	5.68	233		21.33	6.10				
	9‐week Follow‐up	150	59		17.34	7.34	142	61	18.35	6.68	−1.69	−2.99 to −0.39	0.011	0.28
HADS														
Morning chronotype	Baseline	163			11.32	6.46	179		13.33	6.92				
	9‐week Follow‐up	118	72		8.58	6.34	114	64	11.41	7.20	−1.78	−3.03 to −0.54	0.005	0.25
Intermediate chronotype	Baseline	410			13.02	6.91	401		12.97	7.25				
	9‐week Follow‐up	288	70		9.89	7.00	243	61	10.55	6.56	−1.24	−2.05 to −0.41	0.003	0.18
Evening chronotype	Baseline	253			14.56	7.08	233		14.27	7.29				
	9‐week Follow‐up	149	59		11.93	7.13	138	59	12.96	7.19	−0.85	−1.95 to 0.27	0.139	0.12

The difference estimates are results from the baseline‐adjusted linear mixed models (negative values favour dCBT‐I). Percentage (%) is calculated based on response rate between the baseline to the 9‐week follow‐up.

CFQ, Chalder Fatigue Questionnaire; dCBT‐I, digital cognitive behaviour therapy for insomnia; HADS, Hospital Anxiety and Depression Scale; ISI, Insomnia Severity Index; PE, patient education about sleep.

As shown in Table 2, there were significant differences in amount of change from baseline to 9‐week follow‐up scores between dCBT‐I and PE on the ISI for the three chronotype groups, in favour of dCBT‐I. There were significant differences in treatment effects between the three chronotypes on the ISI (three‐way interaction, *p* = 0.023), in favour of morning and intermediate chronotypes (estimated difference between evening and morning type of −1.70, 95% confidence interval [CI]: −2.96 to −0.45, *p* = 0.008, and −1.53, 95% CI: −3.04 to −0.03, *p* = 0.046 between evening and intermediate type).

There were significant differences in amount of change from baseline score to 9‐week follow‐up scores between dCBT‐I and PE on the CFQ for the three chronotype groups, in favour of dCBT‐I. There were no significant differences in treatment effects across chronotypes on the CFQ (three‐way interaction, *p* = 0.488).

There were significant differences in amount of change from baseline score to 9‐week follow‐up scores between dCBT‐I and PE on the HADS for individuals classified as morning or intermediate chronotypes, in favour of dCBT‐I. There were no significant differences in change from baseline score to 9‐week follow‐up between dCBT‐I and PE on the HADS for individuals with evening type chronotype. There were no significant differences in treatment effects between the three chronotype categories on the CFQ (three‐way interaction, *p* = 0.488).

**FIGURE 1:**
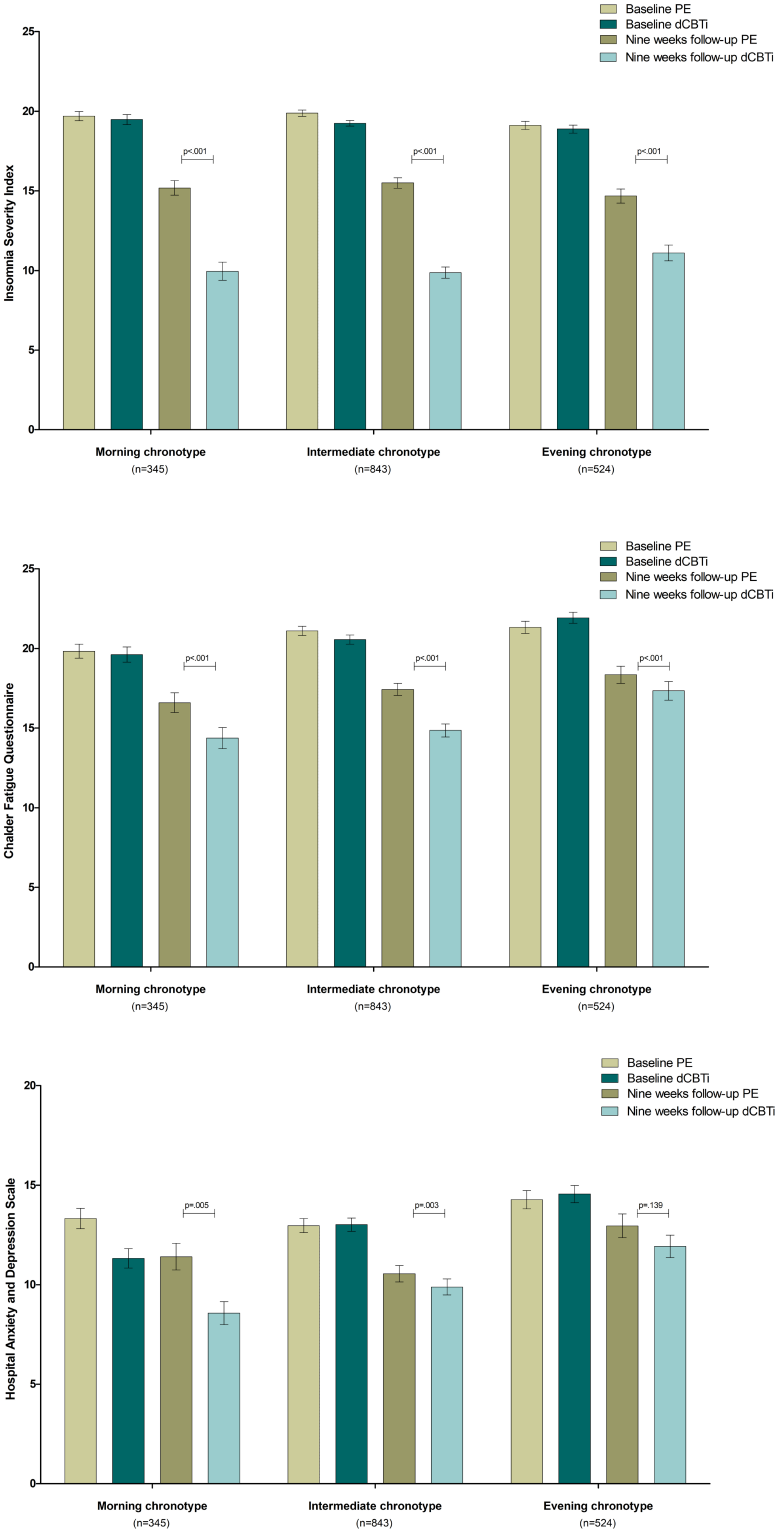
Observed means and standard error (SE) for each group at baseline and at 9‐week follow‐up for chronotypes who were allocated to either digital cognitive behaviour therapy for insomnia (dCBT‐I; *n* = 867) or patient education about sleep (PE; *n* = 853). The *p*‐values represent differences between the groups in change from baseline to 9 weeks (group × time interaction term in the linear mixed model), with Insomnia Severity Index (ISI), Hospital Anxiety and Depression Scale (HADS) and Chalder Fatigue Questionnaire (CFQ) as dependent variables

A visual inspection of the effect sizes (i.e. Cohen's *d*) for the ISI, CFQ and HADS shows that individuals with morning and intermediate chronotypes exhibited the largest effect sizes (Table 2). A visual representation of observed means and standard error is shown in Figure 1.

## 4 DISCUSSION

The main aim of this study was to test whether chronotype, as measured by self‐report at baseline, moderated the effects of dCBT‐I on insomnia severity, psychological distress and fatigue at 9‐week follow‐up. The results from the three‐way interaction between group, time and chronotype indicated that chronotype was a significant moderator of the amount of change in ISI scores with dCBT‐I. Participants who were classified as morning or intermediate chronotype exhibited a significantly greater improvement in their ISI score at follow‐up, compared with PE, with large effect sizes (Cohen's *d* > 1.0), and all groups had significantly greater improvements on the secondary outcome variables when compared with PE, albeit with small to medium effect sizes. These findings demonstrate that self‐reported chronotype is a possible indicator of how well people with insomnia complaints will respond to a dCBT‐I. Yet, it is not possible to say whether this information could be used to select individuals for self‐administered dCBT‐I (as opposed to face‐to‐face CBT‐I), as all chronotypes appear to have an effect of the intervention in the present study. However, it should be noted that we used relatively broad categorizations of morning and evening chronotypes in this study, in which a more fine‐grained differentiation and identification of the extreme chronotypes could have given a different result.

The results of this study indicate that there is a variation in the effect of dCBT‐I across personal preferences in terms of chronotype. We also know from previous research that an individual's preference regarding chronotype is reflected in the sleep difficulties they experience. In particular, individuals with an extreme circadian preference are more likely to have a delayed or advanced timing of the sleep–wake rhythm (Ferrante et al., 2015), and chronotype is correlated with the circadian phase of the endogenous pacemaker (dim light melatonin onset; Liu et al., 2000). We found that chronotype categories differ at baseline sleep measurements. Hence, participants who were classified as evening chronotype exhibited a significantly longer SOL, less WASO, less EMA, better SE, longer TIB and more TST compared with morning or intermediate chronotypes. Furthermore, evening chronotypes report higher levels of fatigue and psychological distress. It is possible that this affects our findings on several interrelated levels.

For one thing, individuals may have difficulty adhering to the sleep restriction regime due to a misalignment of the circadian rhythm of the endogenous pacemaker and desired sleep–wake times. The assigned window available for sleep in a sleep restriction regimen is calculated based on each participant's TST at baseline, and participants chose a rise time for the entire intervention period. As a result, individuals with morning chronotype might have more difficulty staying up until the prescribed bedtime, due to greater difficulties staying up in the evening/night compared with evening chronotypes. In contrast, individuals with evening chronotype might experience more difficulty rising in the morning at the end of the sleep window. Difficulties adhering to sleep restriction could also lead to reduced effectiveness of the intervention. One of the assumed mechanisms of change in CBT‐I is that increasing the build‐up of the sleep‐dependent homeostasis sleep pressure (process S) will result in the individual falling asleep faster and experiencing improved sleep quality (Borbély, Daan, Wirz‐Justice, & Deboer, 2016). However, evening type individuals might experience an underlying mismatch of sleep‐independent circadian phase (process C) and the chosen sleep–wake window. This may result in reduced homeostatic sleep pressure when going to bed and therefore difficulties falling asleep. Further, and on a related note, some participants within the morning or evening chronotype might have an underlying circadian rhythm sleep disorder. Nothing was done in this study to exclude participants who had circadian rhythm sleep problems. It is well known that participants with circadian rhythm sleep disorder might present with similar symptoms as those of people with insomnia (Gradisar et al., 2011), such as prolonged SOL for individuals with a delayed sleep phase disorder or EMA for individuals with advanced sleep phase disorder. Both of which may result in reduced SE and daytime symptoms of sleepiness (Sack et al., 2007). However, participants with a circadian rhythm sleep disorder might also have comorbid insomnia (Sateia, 2014), which could have developed as a consequence of spending long periods of time awake in bed, going to bed earlier and developing more negative cognitions about sleep (Morin & Espie, 2007).

Another aspect that should be considered with regards to our current findings is that previous studies have shown that people with different chronotypes are also different in how they score on other personality traits (Adan et al., 2012). People who are evening chronotypes, for example, will score lower on the trait conscientiousness (i.e. being careful or diligent; Adan et al., 2012), which are qualities that may have an impact on compliance to the principles of the dCBT‐I intervention (and, in turn, the effect of the intervention). Future research should investigate whether individuals with insomnia who are offered CBT‐I who also have an evening chronotype adhere differently to sleep restriction compared with individuals with morning or intermediate chronotypes. Personality traits might also mediate the relationship between chronotype and adherence to CBT‐I. Both individuals with eveningness preferences and a delayed sleep phase disorder show a lower score on conscientiousness compared with individuals with morningness preferences (Adan et al., 2012; Wilhelmsen‐Langeland et al., 2014). This may be indicated by differences in response rates at 9‐week follow‐up between the different chronotypes in our study. Individuals high on conscientiousness generally have higher levels of treatment adherence (Hill & Roberts, 2011). Hence, the level of conscientiousness could mediate the results found in this study through adherence.

Although this study includes a larger sample size and more sophisticated moderator analysis than previous similar publications, we acknowledge that there are several limitations. First, although this study was planned a priori, it represents a secondary analysis of data from a previously published RCT. The power calculation focused on the number of individuals required for the RCT and not on the requirements of the current study. Chronotypes are based on one 5‐items self‐reported measurement. Lastly, this study focuses only on immediate post‐intervention outcomes. We do not know if the findings show further change during extended follow‐up. Also, the RCT did not include a third arm (no intervention), thus we do not know if PE participants respond different than no intervention. We observed that the percentage of missing data at follow‐up for participants in dCBT‐I depends on chronotype, hence data are not MCAR. The linear mixed model is unbiased also under the MAR assumption. Given the limitation, replication and confirmation of our findings is required.

Taken together, the findings suggest that chronotype moderates primary outcomes following dCBT‐I, in favour of morning and intermediate chronotype. However, evening chronotype also showed a significant improvement on insomnia severity and fatigue in favour of dCBT‐I when compared with PE. If replicated, these findings point to potential value in determining chronotype prior to recommending dCBT‐I. These findings might also indicate the need for a more dynamic dCBT‐I that can provide circadian intervention for participants with an evening chronotype. Previous research has shown that individuals with high levels of depression symptoms might profit from combining CBT with additional circadian intervention (Leerssen et al., 2021). Some participants within morning or evening chronotypes might additionally have a circadian sleep disorder or circadian misalignment, and might therefore also be in need of additional intervention. Hence, future research ought to investigate how individuals with different chronotypes respond differently to dCBT‐I, and explore if different circadian phases might moderate dCBT‐I.

## CONFLICT OF INTEREST

LMR report financial or business interests in BeHealth Solutions and Pear Therapeutics, two companies that develop and disseminate digital therapeutics (including by licensing the therapeutic developed) based in part on early versions of the software from the University of Virginia, which is used in the research reported in this article. These companies had no role in preparing this manuscript. LMR is also a consultant to Mahana Therapeutics, a separate digital therapeutic company not affiliated with this research. All other authors declare no competing interests.

## AUTHOR CONTRIBUTIONS

ØV: conceptualization, methodology, investigation, data curation, supervision, project administration, resources, writing – review and editing. KL: conceptualization, methodology, investigation. BS: conceptualization, methodology, investigation, supervision, funding acquisition. SL: methodology, formal analyses. CLV: data curation, investigation. KK: investigation. DV: investigation. LMR: conceptualization, writing – review and editing, resources. AGH: conceptualization, writing – review and editing. TCS: conceptualization. JS: conceptualization, methodology, investigation, supervision, writing – review and editing. HK: conceptualization, methodology, investigation, supervision, project administration, resources, writing – review and editing, funding acquisition. PF: writing – original draft, data curation, investigation.

## Supporting information

Table S1‐S3

## Data Availability

The data that support the findings of this study are available on request from the corresponding author. The data are not publicly available due to privacy or ethical restrictions.
