# Supplementary material for: Digital cognitive behaviour therapy for insomnia (dCBT‐I): Chronotype moderation on intervention outcomes
Source: J Sleep Res. 2022 Feb 27;31(5):e13572. doi: 10.1111/jsr.13572 (PMC9787033; doi:10.1111/jsr.13572)
Supplement: Supplementary file 1 — Table S1‐S3 [file JSR-31-e13572-s001.docx]

| **Supplement Table 1.** Remitters and responders to dCBT-I, dCBT-I (n=867) and PE (n=853). Remitters includes participants that have ISI score less than 8 points, at 9-week follow-up. Responders includes participants that have decrease ISI of >8 points. | | | | | | | | |
| --- | --- | --- | --- | --- | --- | --- | --- | --- |
|  | dCBT-I | | | | Patient Education (PE) | | | |
|  | | n | % |  | | n | % |  |
| **Remitters** | |  |  |  | |  |  |  |
| Morning chronotype | | 42 | 35% |  | | 6 | 5% |  |
| Intermediate chronotype | | 107 | 37% |  | | 15 | 6% |  |
| Evening Chronotype | | 53 | 34% |  | | 19 | 12% |  |
|  | |  |  |  | |  |  |  |
| **Responders** | |  |  |  | |  |  |  |
| Morning chronotype | | 74 | 62% |  | | 22 | 19% |  |
| Intermediate chronotype | | 167 | 58% |  | | 56 | 22% |  |
| Evening Chronotype | | 79 | 51% |  | | 34 | 22% |  |
|  | |  |  |  | |  |  |  |

| **Supplement Table 2.** Primary and secondary outcomes at 9-week follow-up assessment for participants grouped into five different circadian preferences who were allocated to either dCBT-I (n=867) or PE (n=853). | | | | | | | | | | |
| --- | --- | --- | --- | --- | --- | --- | --- | --- | --- | --- |
|  | dCBT-I | | | Patient Education (PE) | | | Adjusted mean difference | | | |
|  | n | Mean | SD | n | Mean | SD | Estimate | 95% CI | P-value | Cohen’s d |
| **Insomnia Severity Index (ISI)** |  |  |  |  |  |  |  |  |  |  |
| Definitely Morning | 10 | 14.40 | 7.46 | 10 | 14.00 | 4.97 | 0.36 | -3.50 to 4.22 | .855 | -0.09 |
| Moderately Morning | 116 | 9.64 | 6.23 | 108 | 15.26 | 4.91 | -5.75 | -6.93 to -4.58 | <.001 | 1.47 |
| Neither Type | 300 | 10.04 | 6.11 | 259 | 15.45 | 5.31 | -5.42 | -6.16 to -4.67 | <.001 | 1.38 |
| Moderately Evening | 121 | 10.89 | 6.23 | 112 | 14.76 | 5.31 | -4.22 | -5.37 to -3.07 | <.001 | 1.08 |
| Definitely Evening | 36 | 12.50 | 6.16 | 45 | 14.84 | 6.13 | -2.02 | -4.07 to 0.03 | .054 | 0.52 |
|  |  |  |  |  |  |  |  |  |  |  |
| **Chalder Fatigue Scale (CFQ)** |  |  |  |  |  |  |  |  |  |  |
| Definitely Morning | 10 | 16.30 | 3.95 | 10 | 16.70 | 5.76 | -0.40 | -5.36 to 4.56 | .874 | 0.07 |
| Moderately Morning | 113 | 14.22 | 6.52 | 107 | 16.68 | 7.24 | -2.88 | -4.40 to -1.37 | <.001 | 0.48 |
| Neither Type | 297 | 14.90 | 6.97 | 255 | 17.53 | 6.47 | -2.59 | -3.55 to -1.63 | <.001 | 0.44 |
| Moderately Evening | 121 | 16.88 | 6.82 | 112 | 18.47 | 7.35 | -2.00 | -3.48 to -0.52 | .008 | 0.34 |
| Definitely Evening | 33 | 18.46 | 7.86 | 44 | 18.75 | 7.26 | -0.65 | -3.36 to 2.06 | .639 | 0.11 |
| **Hospital anxiety and depression scale (HADS)** |  |  |  |  |  |  |  |  |  |  |
| Definitely Morning | 10 | 8.60 | 8.31 | 10 | 8.10 | 9.74 | -1.96 | -6.16 to 2.25 | .361 | -0.28 |
| Moderately Morning | 110 | 8.65 | 7.27 | 105 | 11.73 | 6.31 | -1.77 | -3.07 to -0.47 | .008 | 0.25 |
| Neither Type | 295 | 9.96 | 6.98 | 252 | 10.48 | 5.99 | -1.23 | -2.05 to -0.41 | .003 | 0.18 |
| Moderately Evening | 120 | 11.75 | 6.96 | 110 | 12.95 | 6.77 | -1.38 | -2.64 to -0.12 | .032 | 0.20 |
| Definitely Evening | 33 | 12.58 | 8.57 | 42 | 13.45 | 6.62 | 0.95 | -1.38 to 3.28 | .424 | 0.14 |
| Notes.  The difference estimates are results from the baseline-adjusted linear mixed models (negative values favor dCBT-I). | | | | | | | | | | |

| **Supplement Table 3.** Description and completion rates for dCBT-I sessions during the intervention period dCBT-I (n=867) | | | | |
| --- | --- | --- | --- | --- |
|  | Description of session | Number of participants in dCBT-I group who completed session. | | |
|  |  | Morning chronotype (*n*=164) | Intermediate chronotype (*n*=419) | Evening chronotype (*n*=267) |
| Core 1: overview | Reviews the nature of insomnia and how the program works; the participants identify their sleep problems and set up personal treatment goals | 146 (89%) | 367 (88%) | 217 (81%) |
| Core 2: Behavior and sleep 1 | Focuses on how behavioral changes can improve sleep, with a special emphasis on sleep restriction | 127 (77%) | 310 (74%) | 186 (70%) |
| Core 3: Behavior and sleep 2 | Focuses on how behavioral changes can improve sleep, with a special emphasis on stimulus control | 114 (70%) | 274 (65%) | 158 (59%) |
| Core 4: Sleep and thoughts | Focuses on addressing and changing beliefs and thoughts that might impair sleep (eg, excessive worrying about the possible consequences of insomnia) | 107 (65%) | 249 (59%) | 131 (49%) |
| Core 5: Sleep hygiene | Teaches about lifestyle and environmental factors that might interfere with sleep (eg, caffeine and nicotine intake, and electronic media use in bed) | 95 (58%) | 228 (54%) | 109 (41%) |
| Core 6: Relapse prevention: | Focuses on integrating the behavioral, educational, and cognitive components from the former cores to develop strategies to avoid future episodes of poor sleep from developing into full-blown chronic insomnia | 87 (53%) | 210 (50%) | 90 (34%) |
|  |  |  |  |  |
